# Strategies to Limit Cognitive Impairments under Sleep Restriction: Relationship to Stress Biomarkers

**DOI:** 10.3390/brainsci12020229

**Published:** 2022-02-07

**Authors:** Danielle Gomez-Merino, Catherine Drogou, Eden Debellemaniere, Mégane Erblang, Rodolphe Dorey, Mathias Guillard, Pascal Van Beers, Melanie Thouard, Robin Masson, Fabien Sauvet, Damien Leger, Clément Bougard, Pierrick J. Arnal, Arnaud Rabat, Mounir Chennaoui

**Affiliations:** 1 Unité Fatigue et Vigilance, Institut de Recherche Biomédicale des Armées (IRBA), 91223 Bretigny-sur-Orge, France; catherine.drogou@gmail.com (C.D.); eden.dbm@gmail.com (E.D.); megane.erblang@gmail.com (M.E.); rodolphe.dorey@gmail.com (R.D.); mathias.guillard55@gmail.com (M.G.); pavanbeers@gmail.com (P.V.B.); fabien.sauvet@gmail.com (F.S.); clement.bougard@live.fr (C.B.); arnaud.rabat.irba@gmail.com (A.R.); 2 VIgilance FAtigue SOMmeil et Santé Publique, Université de Paris, 75004 Paris, France; damien.leger@aphp.fr; 3 Dreem SAS, 75009 Paris, France; pierrick.arnal@gmail.com; 4 Ecole du Val de Grace, 75005 Paris, France; melanie.thouard@gmail.com (M.T.); robin.83@hotmail.fr (R.M.); 5 Centre du Sommeil et de la Vigilance, APHP, Hôpital Hôtel-Dieu, 75004 Paris, France

**Keywords:** sleep-deprived, recovery, auditory EEG slow oscillation, relaxation technique, cognition, stress biomarkers

## Abstract

Adding relaxation techniques during nap or auditory stimulation of EEG slow oscillation (SO) during nighttime sleep may limit cognitive impairments in sleep-deprived subjects, potentially through alleviating stress-releasing effects. We compared daytime sleepiness, cognitive performances, and salivary stress biomarker responses in 11 volunteers (aged 18–36) who underwent 5 days of sleep restriction (SR, 3 h per night, with 30 min of daily nap) under three successive conditions: control (SR-CT), relaxation techniques added to daily nap (SR-RT), and auditory stimulation of sleep slow oscillations (SO) during nighttime sleep (SR-NS). Test evaluation was performed at baseline (BASE), the fifth day of chronic SR (SR5), and the third and fifth days after sleep recovery (REC3, REC5, respectively). At SR5, less degradation was observed for percentage of commission errors in the executive Go–noGo inhibition task in SR-RT condition compared to SR-CT, and for sleepiness score in SR-NS condition compared both to SR-CT and SR-RT. Beneficial effects of SR-RT and SR-NS were additionally observed on these two parameters and on salivary α-amylase (sAA) at REC3 and REC5. Adding relaxation techniques to naps may help performance in inhibition response, and adding nocturnal auditory stimulation of SO sleep may benefit daytime sleepiness during sleep restriction with persistent effects during recovery. The two strategies activated the autonomic nervous system, as shown by the sAA response.

## 1. Introduction

In industrialized countries, professionally active populations (e.g., healthcare professions, drivers, soldiers) usually face a routinely reduced sleep time as compared to the amount required for optimal functioning [1,2,3]. This chronic sleep restriction leads to daytime sleepiness [4], cognitive deficits, and impaired response of the stress-related activities of the hypothalamo-pituitary (HPA) axis and the autonomic nervous system (ANS) [5,6]. With time, these deficits increased the risk of developing cardiovascular diseases in the adult general population, as well as induced high risk-behavior in military service members [7,8]. Given the seriousness of the consequences of chronic sleep deprivation, the countermeasure of napping is currently recommended by the medical profession and practiced by shift workers [9] and soldiers to reduce fatigue and limit performance degradation related to sleep/wake disruptions [2,10]. In young healthy individuals (men and women), a 2 h midafternoon nap after one night of sleep loss restores, to a significant degree, alertness and tends to improve, to a lesser degree, performance in the psychomotor vigilance task (PVT) [11]. A 30 min nap decreased sleepiness and salivary cortisol levels induced by sleep restriction [12]. In addition, pharmacological countermeasures using the combination of napping with modafinil or caffeine were proven to improve vigilance and performance in simulated operational military or shift-work conditions as early as the 1990s [13,14]. However, they both induce side effects and do not directly compensate all neurocognitive effects of sleep debt. Recent further considerations have emphasized the interest of relaxation techniques for the optimization of nap sleep or auditory stimulation of EEG slow oscillations (SO) during nocturnal slow wave sleep (SWS) using an ambulatory dry-EEG device [15,16]. In healthy young subjects with no sleep debt, auditory closed-loop stimulation of sleep SO for one night has been shown to improve memory consolidation [17,18], to further reduce SWS-related cortisol levels [19], and to enhance slow-wave activity in association with a reduction in evening-to-morning change of cortisol levels and indices of sympathetic activity [20]. A recent study showed beneficial effects of acoustic SO sleep stimulation during two nights on next day daytime alertness and performance on the psychomotor vigilance task (PVT) in chronically short sleepers [21]. Meanwhile, relaxation techniques were shown to improve the coping styles in care nurses and also to improve short-term memory in 10–11-year-old primary school children [22,23]. We showed the deepening of short nap when adding relaxation techniques (including progressive muscle relaxation and hypnosis) [15]. However, there are no data to our knowledge on relaxation techniques added to nap in sleep-deprived healthy adults.

We asked whether optimizing daytime naps or nighttime sleep in young, healthy but sleep-deprived subjects could facilitate recovery favorable to operational capacity. Our objective was to evaluate daytime sleepiness, cognitive performance, and stress biomarkers responses after 5 days of sleep restriction (3 h per night with a 30 min early afternoon nap) under the condition of adding relaxation techniques during daytime nap or auditory SO stimulation during nighttime sleep. Responses were additionally compared at the third and fifth days after sleep recovery. Salivary levels of cortisol and the reliable biomarkers of the catecholamine release, α-amylase (AA) and chromogranin-A (CgA) levels, were determined (reviewed by [24]). The laboratory sleep restriction protocol including a daily 30 min nap is as close as possible to what soldiers practice when they are sleep-deprived, which was induced in order to ensure that our results can be applied to the real-world operational context of soldiers.

## 2. Materials and Methods

### 2.1. Participants

Thirteen participants (7 men and 6 women, aged 18–36 years) with moderate morning chronotypes were included in this cross-over study. Routine surveys and a medical interview with a physician ensured that they had no history of neurological, psychiatric, or endocrine disease, including any sleep disorder. They reported having good sleep quality on the Pittsburgh sleep quality index (PSQI) (3.1 ± 0.5) [25] and being not symptomatic (5.6 ± 1.0) in the Hospital Anxiety and Depression Scale (HADS) [26]. They were non-smokers or light smokers (two cigarettes were allowed per day at a fixed time). None of the participants had participated in any shiftwork or had travelled across more than one time zone within the previous 4 weeks. They were asked to follow a regular sleep/wake rhythm, even during weekends, for at least two weeks prior to the experiment with 7–10 h per night and no daytime naps. To this purpose, we assessed their sleep and wake patterns with a sleep agenda and a wrist-actimeter (Actiwatch^TM^; Cambridge Neurotechnology, Cambridge, UK). All procedures were in accordance with the Declaration of Helsinki. The ethics committee of the Hotel Dieu—Ile de France 1 (Paris) and the drug safety national agency (Agence nationale de sécurité du médicament et des produits de santé) approved the protocol (N°IDRCB: 2016-A01165-46). Accordingly, participants gave their informed written consent before participating in the experiment.

### 2.2. Experimental Design

**Figure 1:**
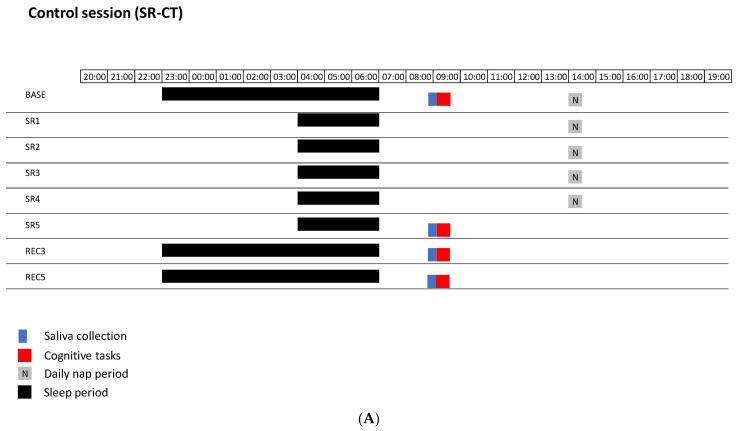

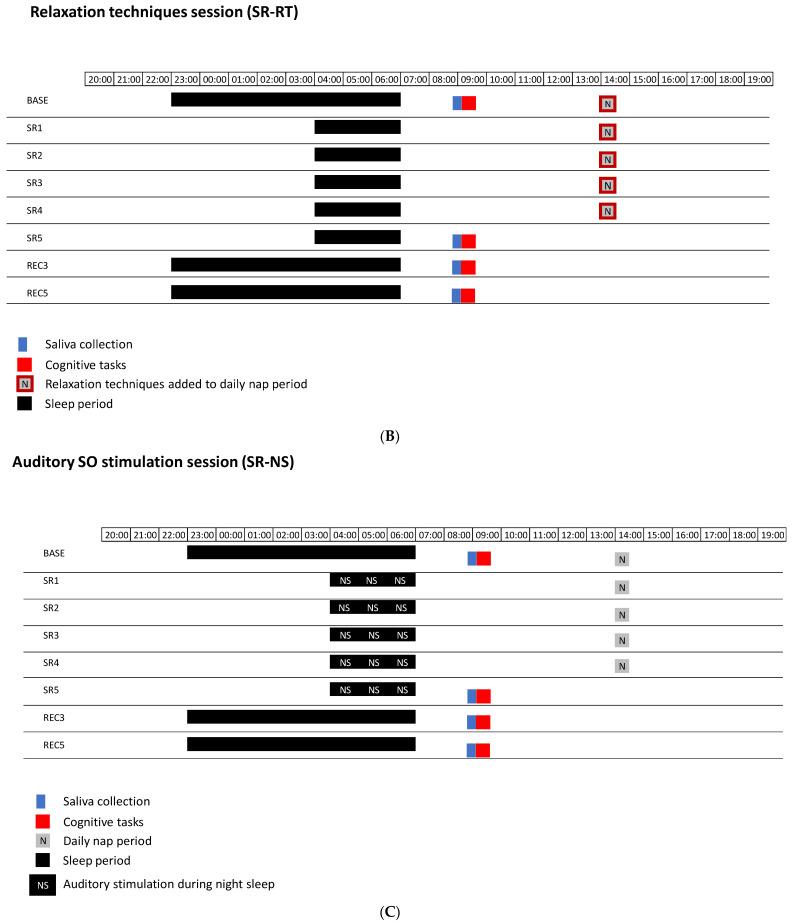
The three sessions of in-laboratory protocol of 5 days of sleep restriction with 30 min of early afternoon nap (SR): the control session (SR-CT) (**A**), the session with relaxation techniques added to SR (SR-RT) (**B**), and the session with auditory SO stimulation added during nighttime sleep (SR-NS) (**C**). Each session included a baseline (BASE) day with 8 h time-in-bed (TIB), 5 days of chronic sleep restriction (SR, with 3 h TIB with 30 min nap), followed by 5 days of sleep recovery (REC) with 8 h TIB.

The protocol included three successive and non-counterbalanced sessions, with a wash-out of six weeks, of 5 days of sleep restriction with 30 min of early afternoon nap (SR): a control session (SR-CT), a session with relaxation techniques during nap (SR-RT), and a session with auditory SO stimulation during nocturnal sleep (SR-NS). All participants participated in each session. For each session, participants spent 12 days in the sleep laboratory of the Hôtel-Dieu Hospital. Each session included a habituation day (HAB) with 8 h of time in bed (TIB) (from 23:00 to 07:00), a baseline day (BASE) with 8 h TIB (from 23:00 to 07:00), a 5 day restricted-sleep period (3 h TIB during the night, from 04:00 to 07:00) with a 30 min nap at 14:15 (SR), and a 5 day recovery period (REC) (8 h TIB during the night, from 23:00 to 07:00) (Figure 1). The SR-CT session was free from relaxation techniques during the early afternoon nap and free from auditory SO stimulation during the 5 nights of restricted sleep. The SR-RT session was the addition of relaxation techniques to facilitate the 30 min early afternoon nap without nocturnal auditory sleep SO stimulation. The SR-NS session encompassed nocturnal auditory sleep SO stimulation delivered by the Wireless Dreem Device (in the form of a headband) during the 5 nights of restricted sleep without relaxation technique during the early afternoon nap. Saliva samples were collected at 08:30 in BASE day, after five nights of sleep restriction (SR5), and after three and five nights of sleep recovery (REC3 and REC5). The sleepiness questionnaire and cognitive tasks were administered after saliva sampling on the same days.

Throughout the entire session, light level, ambient heat, water temperature of showers, and food intake were standardized, controlled, and kept stable. Participants were all living together in the sleep lab where quiet activities (e.g., reading, parlor game, TV) were authorized except for the 30 min preceding tasks. During all the sessions in the sleep laboratory, including the tests, actimetry activity was recorded to confirm that all participants stayed awake during the wakefulness period. At least two investigators were systematically present. The 30 min naps took place in a collective room on gym mats.

To sleep, participants were allowed to reach their individual rooms 10 min prior to sleep time (23:00 in BASE and REC days and 4:00 in SR days). They were gently awakened by an experimenter in the morning.

### 2.3. Methods

#### 2.3.1. Procedure for Relaxation Techniques in the SR-RT Session

A military instructor who had received full training in relaxation techniques, hypnosis, and mental skill training administered relaxation techniques during the whole nap duration [15,27]. These techniques involved progressive muscle relaxation, hypnosis, and paradoxical interventions, being part of the mental skill training developed by the French armed forces and utilized since 1993.

#### 2.3.2. Procedure for Auditory Sleep SO Stimulations in the SR-NS Session

Stimulations were delivered by the WDD headband during SR nights [14,17]. It consisted in trains of 2 consecutive clicks of 40 dB 100 ms pink noise played in the ascending phase of the SO after 10 min of stable N3 (e.g., no micro-arousal or sleep change). Stimulation automatically stopped when a micro-arousal, a movement, or a sleep change was detected.

#### 2.3.3. Outcome Measurements and Study Instruments

*Nighttime and nap sleep assessment.* In order to assess sleep architecture, we equipped participants from their arrival to their departure (i.e., from the habituation day (HAB) to REC5) with a miniaturized polysomnography (PSG) recordings (Actiwave, CamNtech LtD; Cambridge, UK), including 3 EEG channels (F3, C3, O1) referenced to M2 and described in our previous studies [5,15]. One electrooculogram (EOG), two electromyogram (EMG), and two electrocardiogram (ECG) channels were hooked up during sleep time to prevent skin irritation. Ag–AgCl electrodes were used, and impedances were regularly checked (every morning, after lunch and prior sleep) and kept below 5 kΩ for EEG electrodes and below 10 kΩ for EOG and EMG electrodes. Signals were sampled at 128 Hz and filtered between 0.3 and 70 Hz. The set-up has been completely removed for days with a shower, for a maximum of 30 min, to avoid any risk of falling asleep. PSG recordings were scored by trained sleep researchers in accordance with the American Academy of Sleep Medicine (AASM) criteria using the SOMNOLOGICA software (TM, Medcare; Reykjavik, Iceland). The nighttime sleep parameters such as the total sleep time (TST) and non-rapid eye movement (NREM) (N1, N2, and N3 sleep stages) and rapid eye movement (REM) sleep stages were determined for BASE, SR1, SR2, SR3, SR4, SR5, REC1, REC2, REC3, REC4, and REC5 nights. The nap sleep parameters were determined for the five days of sleep restrictions and were TST; time-in-bed (TIB); wake after sleep onset (WASO); and N1, N2, N3, and REM sleep stages.

*Subjective levels of sleepiness.* They were assessed each day at 8:45 using the Karolinska Sleepiness Scale (KSS). This scale consists of 9 scores from 1 (extremely alert) to 9 (extremely sleepy, falls asleep all the time) [28].

*Sustained attention*. We utilized a computer-based version of the 10 min psychomotor vigilance task (PVT). This test is easily reproducible, the number of trials per session reduces the hazard bias, and the data are simple enough to be processed efficiently. Subjects were asked to respond by clicking the left mouse button to the appearance of a visual stimulus (a millisecond counter) as quickly as possible without making false starts. The inter-stimulus interval, defined as the period between the last response and the appearance of the next stimulus, varied randomly from 2 to 10 s. The number of PVT lapses of attention is defined as reaction time > 500 ms. The reaction time (RT) in milliseconds for a 1 s period and PVT response was regarded valid if RT was ≥100 ms. Results are expressed as the number of lapses and speed (1/reaction time (RT), also called reciprocal response time) [29].

*Inhibition capacity.* We utilized a computer-based version of the Go/noGo executive task. In this test, participants were required to respond or not to a stimulus on a screen. After the appearance of a fixation cross in the center of the screen for 500 ms, an arrow appeared in the center of the screen for 1 s. Depending on the test instruction, participants had 2 s to respond when the arrow pointed out on the right (“Go” response) and not to respond when it pointed on the left (“noGo“ response). The proportion was always as follows: 67% of “Go” trials and 33% of “noGo” trials. Participants had to 2 s to respond, and their response was directly followed by a new trial in order to determine the capacity to consciously inhibit non-relevant automated responses (inhibition process). The total duration of the task was around 7 min 30 s. Performance of the task was assessed by calculating number of commission errors (ratio) [5,30].

*Working memory capacity.* The 10 min 2-back working memory test was used. The subject was presented with a sequence of letters on the computer screen. The task consisted of indicating (by clicking on the mouse) when the letters matched the one from *n* steps earlier. In our task, our load marker (i.e., *n*) was 2, meaning that they had to click when a presented letter was identical to the one presented two times ago [31]. Results are expressed as percentage of correct responses.

*Saliva biomarkers concentrations.* Participants were asked to gently brush their teeth and to refrain from drink and food intake 30 min prior saliva sampling. They also had to stay still 5 min prior it. The saliva samples analyzed here were collected by passive drool at 08:30 in BASE, SR5, REC3, and REC5. They were directly centrifuged at 3000× *g* for 20 min at 4 °C. Aliquots were taken and stored frozen at −80 °C. They were assayed using an ELISA kit (Salimetrics kit for cortisol; Yanaihara kit for chromogranin-A (CgA)). The salivary α-amylase (sAA) activity was assayed by enzymology with the IBL kit. Assays were made in duplicate, and intra-/inter-assay coefficients of variations (CVs) and the analytical range of sensitivity were of 3/3%, 0.33–83 nmol for cortisol; 10.5/13.3%, 0.14–33.33 pmol/mL for CgA; and 3.7/6.2%, 0–400 UI/mL for sAA.

*Data analyses and statistics*. Data were normalized relative to their baseline value (BASE). Continuous variables were expressed as follows: Normalized value = Value (D Day) − Value (BASE).

All statistical analyses were conducted using Statistica software version 7.1 (Statistica StatSoft, Inc., Tulsa, OK, USA). ANOVAs (Condition × Day, i.e., 3 conditions (SR-CT, SR-RT, SR-NS) and 4 days (BASE, SR5, REC3, REC5) were performed on sleepiness, cognitive parameters, and biological parameters. When the interaction was significant, simple effects were assessed. In the case where the interaction was non-significant, main effects were examined. When the ANOVA revealed significant interactions or main effects, LSD Fisher post hoc tests were used to identify differences. Statistical significance was set at *p* < 0.05. In addition, we performed Pearson’s correlations analysis between KSS score, cognitive tests results, and salivary biological parameters (absolute values) with a statistical significance adjusted to *p* < 0.01.

## 3. Results

### 3.1. Participants

Eleven participants out of the 13 who participated in the three sessions were included in the analyses. One participant had to be removed from the analysis because of a low number of stimulations (<100 per night) and another subject because of a configuration error of his headband (only sham stimulations). The final sample consisted of 5 women and 6 men, aged 18–36 years old, with a BMI of 23.8 ± 0.8 kg/m^2^.

### 3.2. Nighttime and Nap Sleep Assessment

For nighttime sleep parameters, results are expressed as a percentage of NREM and each of N1, N2, N3, and REM sleep stages relative to TST. The ANOVA analysis showed significant effects of the day for all these sleep parameters (*p* < 0.001). There was a significant condition (*p* < 0.018) main effect for REM sleep without interaction (*p* = 0.079) (Figure S1A).

Regarding the mean of sleep parameters during nap for the 5 days of sleep restriction, there was significant day and condition main effects for TST (*p* < 0.001 and *p* < 0.013, respectively), WASO (*p* < 0.017 and *p* < 0.003, respectively), and N1/TST (*p* < 0.001 and *p* < 0.030, respectively), without significant interactions. The TST increased while N1/TST decreased from SR1 to SR5. For N2/TST and N3/TST, there were day main effects (*p* < 0.036 and *p* < 0.001, respectively); the N3/TST sleep increased from SR1 to SR5 (Figure S1B).

### 3.3. Subjective Sleepiness (KSS Score)

**Figure 2:**
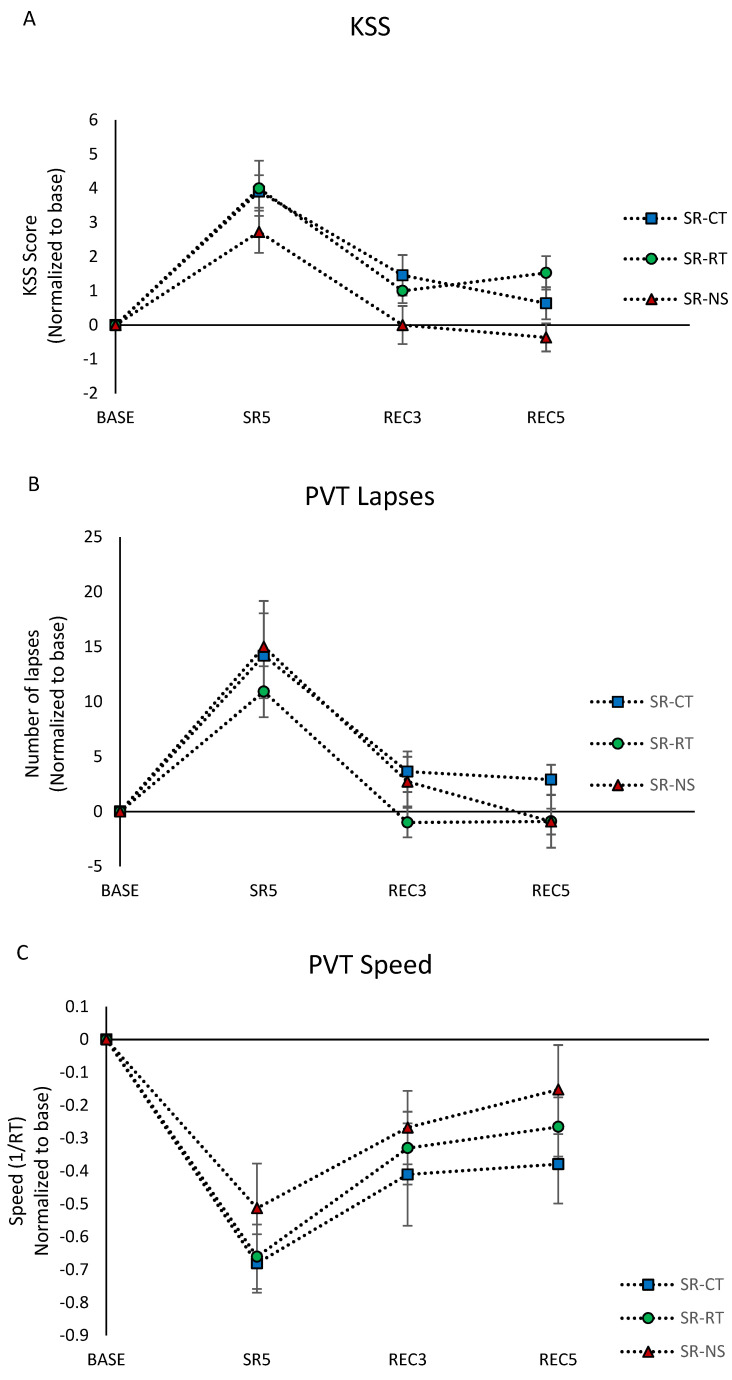
(**A**) Morning KSS scores. (**B**) Number of PVT lapses. (**C**) PVT speed. All are normalized as compared to the baseline day (BASE) and averaged across participants in the control (SR-CT—blue), relaxation techniques (SR-RT—green), and night stimulation of EEG slow oscillations (SR-NS—red) conditions.

The ANOVA analysis showed significant day and condition effects without significant interaction (Table 1). In detail the KSS scores were significantly increased at SR5, REC3, and REC5 compared to the BASE day (Figure 2A). The significant condition main effect (F = 5.11, *p* < 0.05) showed lower levels in the SR-NS condition compared with SR-CT and SR-RT (*p* = 0.02 and *p* = 0.01, respectively).

### 3.4. Cognitive Performances

#### 3.4.1. Sustained Attention

Sustained attention was assessed at 08:45 using the 10 min PVT. Regarding the number of lapses (reaction time > 500 ms) and speed, we observed a main day effect and no significant condition effect or condition × day interaction (Table 1). The number of lapses in SR5 increased significantly by an average of 15 compared to the BASE day, returning to the baseline value after the two recovery nights (Figure 2B). The speed was significantly lower at SR5, REC3, and REC5 compared to the BASE day (Figure 2C).

#### 3.4.2. Inhibition Capacity

**Figure 3:**
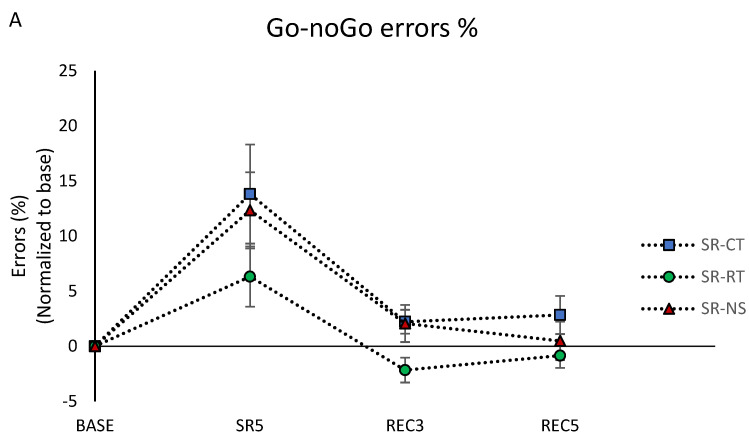

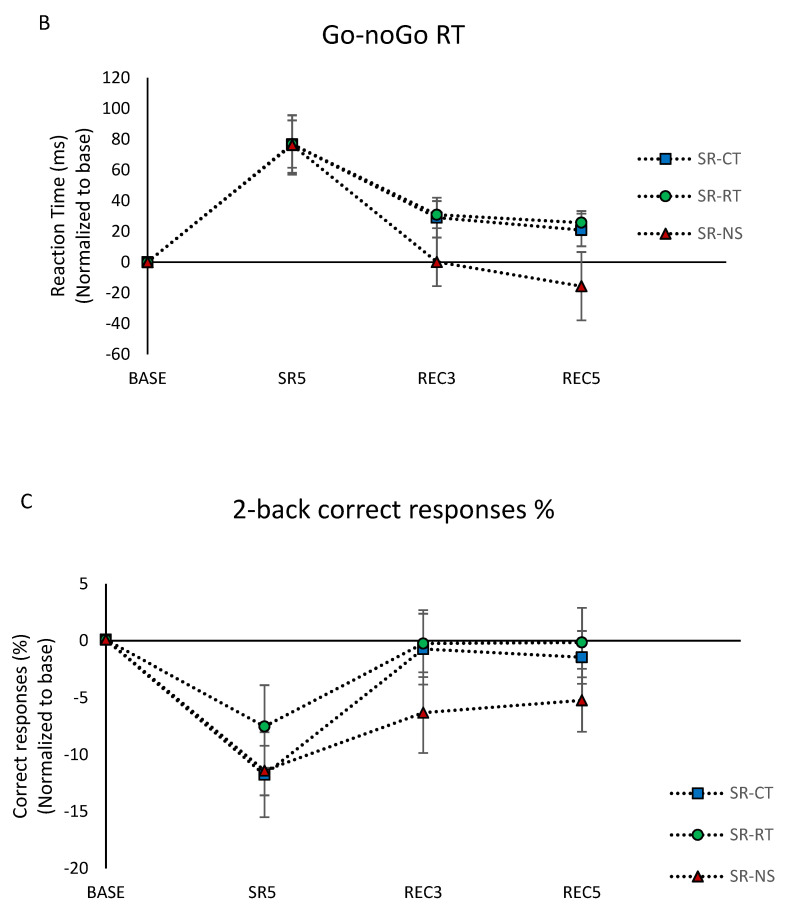
(**A**) Percentage of Go–noGo commission errors. (**B**) Go–noGo reaction time. (**C**) Percentage of 2-back correct responses. All are normalized as compared to the baseline day (BASE) and averaged across participants in the control (SR-CT—blue), relaxation techniques (SR-RT—green), and night stimulation of EEG slow oscillations (SR-NS—red) conditions.

Inhibition capacity was assessed at 8:55 using the 10 min Go–noGo test, right after the PVT. For the percentage of commission errors (“noGo responses”), we observed significant day and condition main effects without interaction (Table 1). The percentage of commission errors increased in SR5 compared with the BASE day, returning to baseline values after the two nights of recovery (Figure 3A). The significant condition main effect (F = 5.03, *p* < 0.05) showed lower levels in the SR-RT condition compared with SR-CT and SR-NS (*p* = 0.007 and *p* = 0.04, respectively).

With respect to reaction time (RT) at Go–noGo, we observed a significant day effect but no condition effect or condition × day interaction (Table 1). Reaction time increased in SR5 compared with the BASE day, returning to baseline values after the two recovery nights (Figure 3B).

#### 3.4.3. Working Memory Capacity

Working memory capacity was assessed using the 2-back task after the PVT and the Go–noGo (i.e., at 9:05). Regarding the percentage of correct responses, we only observed a significant main day effect with a significant decrease of the percentage in SR5 and REC3 compared with the BASE day (Table 1, Figure 3C).

### 3.5. Salivary Biomarkers Concentrations

**Figure 4:**
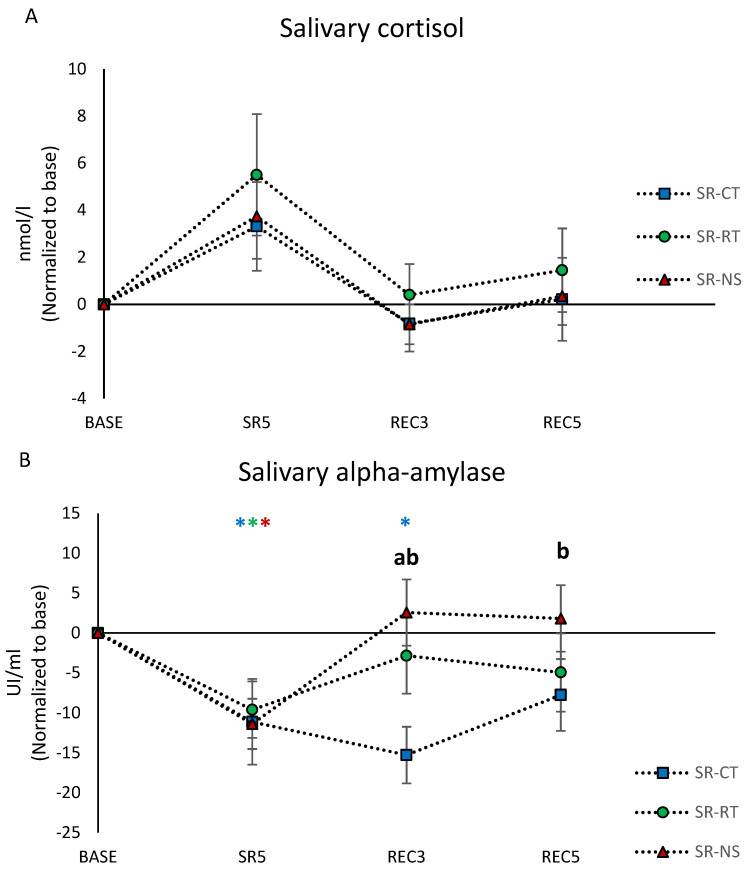

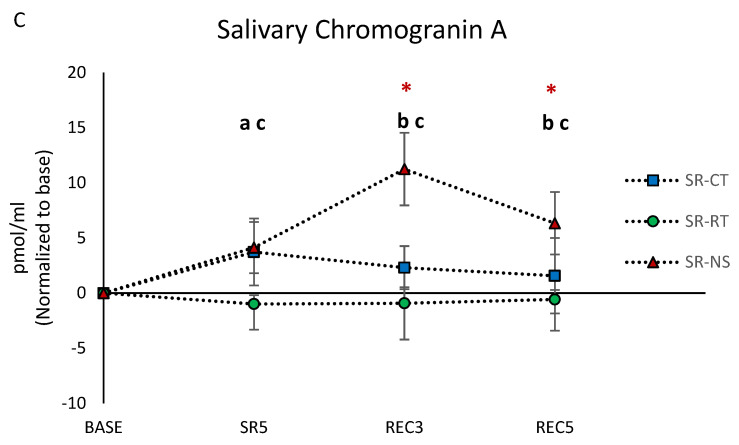
(**A**) Morning salivary concentrations of cortisol. (**B**) α-amylase (sAA). (**C**) Chromogranin-A (CgA). All are normalized as compared to the baseline day (BASE) and averaged across participants in the control (SR-CT—blue), relaxation techniques (SR-RT—green), and night stimulation of EEG slow oscillations (SR-NS—red) conditions. * indicates a statistical significant difference as compared to the baseline (BASE); (a) indicates a statistical significant difference between SR-RT and SR-CT conditions, (b) between SR-NS and SR-CT, (c) between SR-RT and SR-NS.

The salivary concentrations of cortisol did not show a condition or condition × day effect (Table 1). However, we observed a main day effect with a significant increase at SR5 compared to the BASE day (Table 1, Figure 4A).

Significant main day effect and condition × day interaction were found for the salivary alpha-amylase (sAA) concentration (Table 1). The post hoc analysis showed that sAA concentration was significantly decreased at SR5 compared with the BASE day, and the decrease persisted in REC3 in the SR-CT condition (Figure 4B). The sAA concentration in the SR-CT condition was significantly different from the SR-RT and SR-NS conditions at REC3 and from the SR-NS condition at REC5 (Figure 4B).

Significant main day and condition effects and condition × day interaction were found for the salivary concentrations of CgA (Table 1). The CgA concentrations were significantly higher in the SR-NS condition during the two REC days as compared with the BASE day. The concentration of this protein was significantly lower at SR5 in the SR-RT condition compared to SR-CT and SR-NS. They were significantly higher at REC3 and REC5 in the SR-NS condition compared to the SR-CT and SR-RT conditions (Figure 4C).

### 3.6. Correlation Analysis

Table 2 summarizes the correlations between changes over the protocol in KSS score, cognitive responses, and biological parameters. Changes in KSS scores were significantly correlated with cognitive parameters and not with biological parameters. Levels of PVT lapses were positively correlated with Go–noGo errors (GnG E) and reaction times (GnG RT), while they were in negative correlation with PVT speed (PVT S) and 2-back correct responses (2-B CR). The percentage of Go–noGo errors is positively associated to the Go–noGo reaction times and negatively associated to the 2-back correct responses. The sAA values were negatively correlated to PVT lapses and Go–noGo errors and positively with PVT speed. The CgA concentrations were in positive correlation with Go–noGo reaction time and in negative correlation with the 2-back correct responses.

## 4. Discussion

In the present study, we sought to evaluate if adding relaxation techniques during nap or auditory stimulation of EEG slow oscillations (SO) during nighttime sleep is beneficial for daytime sleepiness and cognitive performances during an in-laboratory protocol of sleep restriction including a 30 min early afternoon nap (SR) followed by sleep recovery. This protocol was chosen because it simulates a week of restricted sleep in a naturalistic military operational setting, which classically includes a short nap in the early afternoon [3,10]. To address this problematic, we refrained participants from sleep with a 3 h of sleep opportunity (between 4 and 7 a.m.) for five nights with an early afternoon 30 min nap in three successive conditions: control (SR-CT), relaxation techniques during nap (SR-RT), and auditory EEG SO during night sleep (SR-NS). Parameters were assessed before sleep restriction (BASE), at SR5 corresponding to the end of sleep restriction, and additionally on the third and fifth days (REC3 and REC5) of sleep recovery (8 h per night). Our results showed significant condition and day effects on the sleepiness KSS score and on the executive Go–noGo inhibition task. For biomarkers, there was a significant day main effect and interaction with condition effect for salivary alpha-amylase (sAA), with concentrations returning to baseline by day REC3 in SR-RT and SR-NS conditions, but not in SR-CT.

On the basis of the three conditions, our protocol first affirmed the abundant literature, reporting a strong effect of five days of sleep restriction (i.e., SR5) on daytime sleepiness and cognitive performances (increased number of lapses and reduced speed in the sustained attention PVT task, increased percentage of commission errors and reaction time in the executive inhibition Go-no-go task, reduced percentage of correct responses in the 2-back working memory task) [5,6,32,33,34]. In our study, we evaluated two strategies that could help limit the cognitive deficits induced by a severe sleep restriction protocol (3 h TIB), including an early afternoon nap that did not appear to be sufficient to compensate for the deficits. Indeed, during such severe sleep restriction (3 h TIB), the PVT performance was evidenced to decline continuously across a 7 day period of sleep restriction, with no apparent stabilization of performance [33]. We confirmed that during severe sleep restriction, a daily 30 min early afternoon nap did not eliminate cognitive deficits [35]. Our results also indicated less errors on the executive Go–noGo motor inhibition task at SR5 when adding a relaxation technique during the daily nap, and lower KSS score when adding SO stimulation during the 3 h nighttime sleep. At SR5, there was no beneficial effect of SR-RT or SR-NS on the vigilance PVT (both lapses number and speed) and working memory 2-back tasks. The Go–noGo executive task evaluates capacity to withhold an automatic response, and increased commission errors for noGo stimuli traduced enhanced impulsivity to negative stimuli [5,36,37]. We recently demonstrated the effectiveness of adding RT (involving progressive muscular relaxation and hypnosis) to deepen an afternoon nap in the workplace [15]. In the present study, no significant differences were observed between the three conditions for nap sleep architecture, but we confirmed our previous study regarding differences between participants for their response to RT [15]. In the literature, RT has been shown to reduce stress and fatigue, as well as to improve coping styles in stressed healthcare workers (i.e., intensive care unit nurses) [22,38]. Progressive muscular relaxation alone was found to potentially reduce brain activity in healthy adult men, and the authors concluded that it may be able to induce a cerebral state appropriate for relaxation, concentration, and resistance to local environmental distractions [39]. While there are no data, to our knowledge, on the benefit of RTs on cognitive impairments related to sleep deprivation in healthy adults, they have been shown to increase short-term memory but not sustained attention in 10–11-year-old elementary school children considered normal in terms of distress level [23]. We suggested that adding RT to the daily 30 min nap for five days of severe sleep restriction is beneficial for the ability to retain an automatic response in the Go–noGo executive task, likely through an impulsivity attenuation effect. On the other side, no condition effect was found on the reaction time Go paradigm of the inhibition task, likely because it is not the main response of impulsive action [40].

In comparison, a lower daytime sleepiness score was observed when adding SO stimulation during the 3 h nighttime sleep without any benefit on cognitive responses. It has been suggested that individuals with the greatest accumulation of slow-wave activity (SWA) during chronic sleep loss can best tolerate sleep loss, with the least increase in sleepiness [41,42]. We and other teams have described that delivering sounds phase-locked to slow-wave sleep is able of enhancing SWA in sleep-deprived and non-sleep-deprived young adults, as well as in older adults [16,17,21], with a beneficial interaction on subjective daytime sleepiness and attention (i.e., PVT performance) and the memory consolidation process [17,18,21]. However, no significant differences in sleep architecture between the test night (i.e., SO stimulation) and the control night were demonstrated [17,18], and the authors suggested that SO phase stimulation primarily drove SO activity, without affecting SO initialization processes [17]. Our results confirmed the latter study since the only significant effect of the condition during nighttime sleep was on the percentage of REM sleep relative to total sleep time (TST) but without interaction with the day condition. A recent study showed that acoustic enhancement of SO during a normal night’s sleep (an 8 h sleep opportunity) enhances parasympathetic activity during SWS in healthy young subjects, and these authors suggested that this may have important implications for cardiovascular health and may also have the potential to improve overall physiological homeostasis [20]. As sleep restriction is associated with increased daytime sleepiness and with no change or increase of sympathetic activity [43,44], we suggested that adding SO acoustic stimulation during the 3 h night sleep and for five days may have promoted SWA and the parasympathetic activity, which in turn would have been involved in a change in our participants’ strategy for coping with sleep pressure that would ultimately result in a significant decrease in subjective sleepiness.

Regarding recovery responses after the sleep restriction protocol, our results showed that the daytime KSS and PVT speed did not return to baseline values at REC3 in the control CT and relaxation technique RT conditions, and that the NS condition with overnight SO auditory stimulation is favorable compared with both conditions for the KSS score only as it returns to baseline value. The effect of acoustic SO stimulation during the 3 h nighttime sleep for five nights on daytime sleepiness may explain why PVT speed appears less degraded in this condition compared with the CT and RT conditions. As PVT speed is considered the primary outcome metric and the most sensible parameter for total sleep deprivation (in comparison with reaction time) [29], the difference observed in the SR-NS condition in recovery from severe sleep restriction may be the NS benefit on daytime sleepiness, which is significantly correlated with all cognitive variables.

At REC3, commission errors and reaction time in the Go–noGo inhibition task recovered baseline values in the three conditions of sleep restriction, while correct responses for the 2-back working memory task were still degraded; the values returned to baseline in the CT and RT conditions and not in the NS condition. Thus, in REC3, the beneficial effect of NS observed on the daytime KSS sleepiness score may have improved speed on the PVT vigilance task, without having an effect on accuracy (i.e., the number of correct responses) for the 2-back working memory executive task. To our knowledge, there are no data on the effects of acoustic closed-loop stimulation during sleep on the 2-back working memory task in healthy adults, and no beneficial effects have been found in healthy children [45]. In our study, because participants experienced the SR-NS condition last, it is possible that the negative effect of deprivation on working memory was still pronounced. Regarding commission errors representing the noGo inhibition paradigm, a beneficial effect of RT over the SR-CT condition was observed at REC3 (and REC5), as well as at SR5, suggesting prolonged effects of impulsivity attenuation when RT was added to the midday nap for five days. Maintenance of the beneficial effect of RT was described in the intensive care unit nurses’ study for stress and fatigue score and coping ability (four weeks after the last RT session, which was delivered once a week for a total of eight weeks) [22]. We suggest that it is because our participants were severely sleep deprived that RT retains effects on impulsivity and thus on the number of errors in the executive Go–noGo task.

With respect to the responses of stress biomarkers to the three conditions at SR-5, we confirmed previous works showing decreased sAA and increased cortisol levels (statistically in the SR-RT and SR-NS conditions) after less severe protocols of sleep restriction (4 h per night during 5 or 7 days) [5,46]. Cortisol levels were not statistically increased in the SR-CT condition, which may have been related to the limited number of subjects in this study. The asymmetry between the hypothalamic–pituitary–adrenal (HPA) axis and the autonomic nervous system (ANS) (i.e., higher cortisol vs. lower sAA, respectively) has been previously observed in subjects with chronic psychological stress, with a signature of blunted reactivity of the ANS [47]. We also confirmed a previous study suggesting that sAA levels may be appropriate indicators of performance deficits for two days (50 h) of total sleep deprivation, as the authors demonstrated significant association between sAA measures and performance on a 3 min PVT and a 40 min simulated driving task [48]. sAA is an enzyme secreted by acinar cells in saliva glands, in which innervation by sympathetic nerves via noradrenaline signaling induces protein release, and thus it has been used as a proxy measure of ANS activity during acute and chronic stress [24,49]. Changes in sAA are reported to be more salient than those of cortisol after a mental stress [50]. The CgA is released along with catecholamines from the adrenal medulla and sympathetic nerve endings and is additionally seen as a biomarker of stress [24], but we are not aware of any data on sleep restriction. At SR5, we found no change in sCgA levels, as previously observed at 7 h after total sleep deprivation, whereas in this latest study, we observed an acute increase after a 60 min motorcycle riding session [51]. In our study, SR-RT and SR-NS conditions influenced sAA and CgA levels, particularly in recovery from severe sleep restriction. At REC3, both RT and NS conditions were found to be beneficial for recovering baseline levels of sAA, while CT was not, and the effect was maintained at REC5 in the NS condition as for the KSS score. Both RT and NS stimulation strategies likely had activating effects on the ANS, at least up to REC3, which in contrast remained blunted by sleep restriction in the SR-CT condition. The beneficial activation of ANS observed at REC3 under SR-RT and SR-NS conditions could be associated with the significant effect of RT on Go–noGo errors and return to baseline on PVT speed only in the NS condition, since sAA levels negatively correlated with Go–noGo errors and positively with PVT speed. Relative to CgA levels at REC3 and REC5, they were significantly higher than baseline in the SR-NS condition, and higher than levels in the SR-CT and SR-RT conditions. This CgA response likely reflects a chronic effect of chronic (i.e., 5 nights) SO sleep stimulation to activate the ANS system, which is not necessarily a beneficial effect for WM performance.

The main limitations of our study include sample size and inter-individual variability with influence of gender and timing of saliva sampling. Thus, the normalization of our data in regard to the baseline of each session was a way to limit biases. Another limitation is that the order of the sessions was not counterbalanced, but this was inherent in the experimental constraints of the protocol. The small number of participants included in our study is inherent to the difficulty of enrolling participants and being able to take care of them for a long in-lab study as proposed here. Having participants in the lab instead of a home study presents the advantage of benefitting from a controlled environment with minor changing across sessions (controlled temperature, light, food intake, etc.). The variability of response between our participants may have led to insufficient statistical power. The gender may also have influenced the cognitive and biological responses to sleep restriction [52]. Hormonal concentrations are strongly influenced by circadian rhythmicity. Cortisol was found to peak within the first 30 min after awakening and decrease gradually until “normal” bedtime; sAA showed the opposite profile, and CgA peaked after awakening and quickly decreased to the nadir in the first hour following awakening. Since saliva was collected 90 min after awakening in our protocol, most of the biological markers of interest were at their lowest concentration. It is therefore plausible that it was not the time where the strongest effect would have been seen. However, since cognitive performance is the most impaired in the morning, it was interesting to report biological change close to cognitive assessment. Finally, the fact that the three sessions were successive without being counterbalanced may have created a seasonal (circannual) effect that may have influenced biomarker concentrations, for example. However, although we lack information on the seasonal rhythm of sAA and CgA, it has been shown that there is no such variation for cortisol that might interfere with effects related to other events (e.g., exercise, sleep deprivation) [53].

## 5. Conclusions

In conclusion, we show here the beneficial effects of adding relaxation techniques to a 30 min nap on the Go–noGo inhibition executive task in severely sleep-deprived subjects, which would reflect reduced impulsivity. In comparison, adding auditory closed-loop stimulation of SO during nighttime had beneficial effects on daytime sleepiness. The beneficial effects were observed after five days of sleep restriction and the third and fifth days of sleep recovery. The two strategies probably acted through stimulating effects on the autonomic nervous system, as shown by change of salivary α-amylase levels. However, these findings should be viewed with great caution because although both strategies temporarily improve some sleepiness and cognitive parameters under sleep experimental restriction conditions, we do not know the potential deleterious consequences of using such approaches in the long term. Future studies could be considered to evaluate these strategies under ecological conditions of sleep deprivation and over the long term.

## Figures and Tables

**Table 1:** ANOVA analysis for KSS, PVT, Go–noGo, and 2-back tasks, as well as biological salivary parameters. * indicates a statistically significant effect at *p* < 0.05, ** at *p* < 0.01, *** at *p* < 0.001.

	Condition (C)	Day (D)	C × D
	F_(2,20)_	F_(3,30)_	F_(6,60)_
KSS	5.11 *	30.1 ***	2.12 (*p* = 0.06)
PVT lapses	1.93 (*p* = 0.17)	14.1 ***	1.05 (*p* = 0.41)
PVT speed	0.86 (*p* = 0.44)	28.7 ***	0.58 (*p* = 0.75)
Go–noGo errors %	5.03 *	10.26 ***	2.19 (*p* = 0.06)
Go–noGo reaction time	0.83 (*p* = 0.45)	16.6 ***	1.28 (*p* = 0.28)
2-back correct responses %	0.90 (*p* = 0.42)	11.2 ***	0.86 (*p* = 0.53)
Cortisol	0.55 (*p* = 0.59)	6.60 **	0.15 (0.99)
Alpha-amylase	2.33 (*p* = 0.12)	5.28 **	2.47 *
Chromogranin A	3.50 *	3.52 *	3.52 **

**Table 2:** Pearson correlation analysis (r coefficients and * for *p* < 0.01).

Variable	KSS	PVT L	PVT S	GnG E	GnG RT	2-b CR	Cortisol	sAA		CgA
KSS	1.000	0.522 *	−0.429 *	0.435 *	0.369 *	−0.325 *	0.197	−0.163		0.048
PVT L		1.000	−0.592 *	0.757 *	0.488 *	−0.503 *	0.119	−0.320 *		−0.025
PVT S			1.000	−0.404 *	−0.561 *	0.374 *	−0.155	0.335 *		−0.010
GnG E				1.000	0.490 *	−0.641 *	0.000	−0.239 *		0.090
GnG RT					1.000	−0.534 *	0.184	−0.070		0.286 *
2-b CR						1.000	−0.019	0.065		−0.300 *

## Data Availability

Data can be obtained by request of the corresponding author.

## Supplementary Materials

The following supporting information can be downloaded at: https://www.mdpi.com/article/10.3390/brainsci12020229/s1.
