# Supplementary material for: Strategies to Limit Cognitive Impairments under Sleep Restriction: Relationship to Stress Biomarkers
Source: Brain Sci. 2022 Feb 7;12(2):229. doi: 10.3390/brainsci12020229 (PMC8869873; doi:10.3390/brainsci12020229)
Supplement: Supplementary file 1 [file brainsci-12-00229-s001.zip › brainsci-1526276-supplementary.pdf]

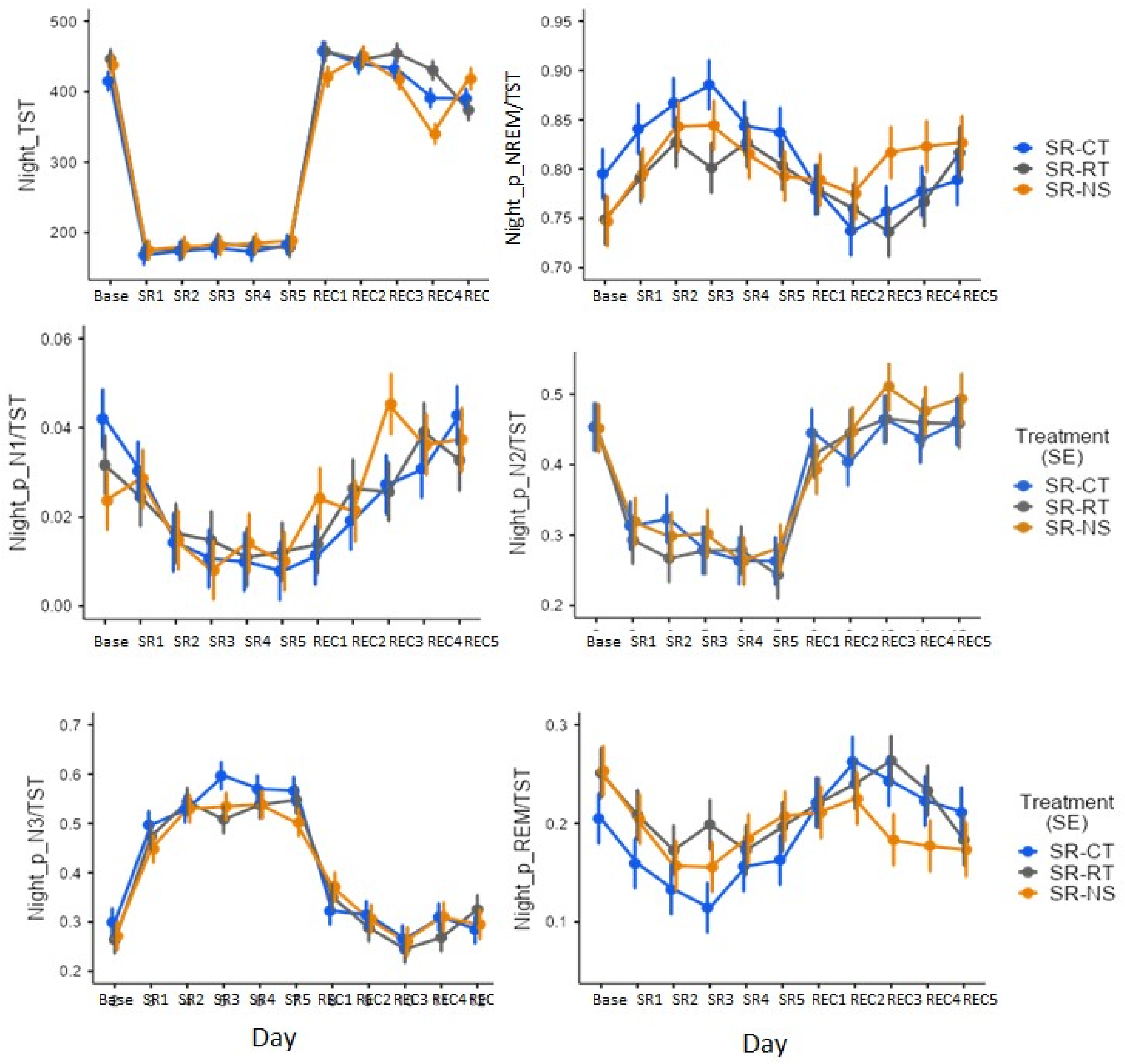

Supplementary Figure S1. Nighttime sleep parameters (expressed in percentage relative to TST during BASE, SR1, SR2, SR3, SR4, SR5, REC1, REC2, REC3, REC4 and REC5 nights. TST: total sleep time, NREM: non-rapid eye movement, REM: rapid eye movement.

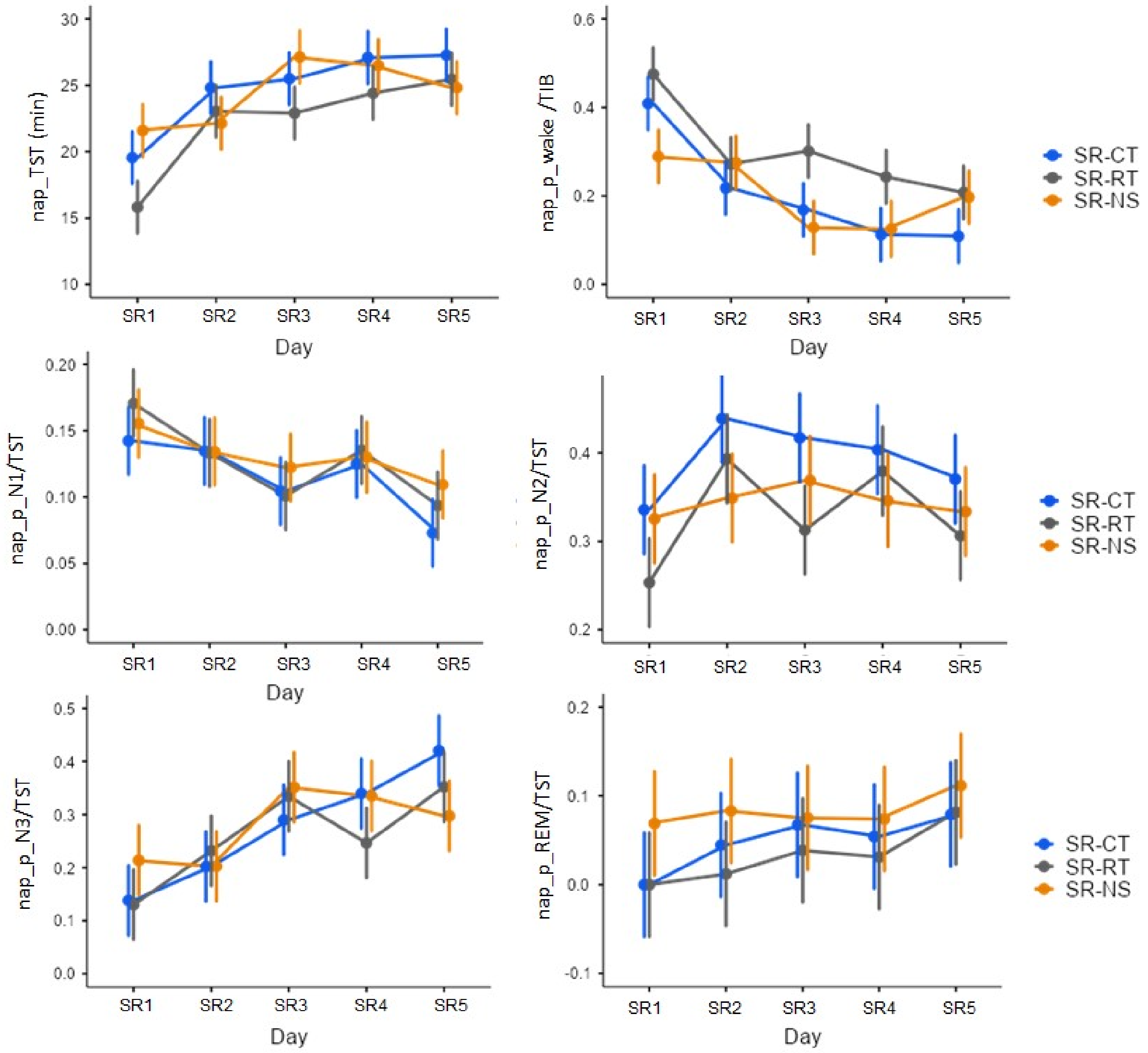

Supplementary Figure S2. Nap sleep parameters (expressed in percentage relative to TST for N1, N2, N3 and REM sleep stages) during SR1, SR2, SR3, SR4, and SR5 days. TST: total sleep time, TIB: time-in-bed, REM: rapid eye movement.
